# Book Notices

**Published:** 1868-11

**Authors:** 


### Book Notices.

A Handbook of Vaccination. By EDWARD C. SEATON, M.D., Medical Inspector to the Privy Council. Philadelphia: J. B. Lippincott & Co. 1868. Pp. 383. Price, $2.25. For sale by S. G. Griggs & Co., Chicago.

This is a very full and convenient treatise on Vaccination, by a very competent author. It contains fourteen Chapters, on the following topics:—Of the natural Cow-Pox; Of the Horse-Pox; Of the Pocks in other Animals; Of the relation of Cow-Pox and Horse-Pox to Human Variola; Of Vaccine, or Cow-Pox in the Human Subject; Of Vaccinating; Of the Maintenance of Lymph-Supply; Of the Conveyance and Storage of Lymph; Of Skill and Success in Vaccinating, and of Insusceptibility; Of alleged degeneration of Lymph, and of recurrence to the Cow; Of the Protection which Vaccination affords; Of Revaccination; Of Stamping-out local Outbreaks of Small-Pox; Of the Objections to Vaccination, and the alleged dangers of the practice.

It is a very instructive volume for the student, and convenient for reference in the library of every practitioner.

---

Constipated Bowels: The various Causes and the different Means of Cure. By S. B. BIRCH, Member of the Royal College of Physicians, of London, etc., etc., etc. From the third London Edition. Philadelphia: Lindsay & Blakiston. 1868. Pp. 181. Price, $1.25.

The general scope of this work is sufficiently indicated by its title. The author discusses the subject with ability, aided by practical experience.

---

Conservative Surgery, in its General and Successful Adaptation, in Cases of Severe Traumatic Injuries of the Limbs, with a Report of Cases. By ALBERT S. WALTER, M.D. Pittsburgh: W. S. Johnston & Co. 1868. Pp. 213—no binding.

This is a monograph, in paper cover, of 213 pages. After a few pages of introductory remarks on the value of *Conservatism* in Surgery, the remainder of the work is occupied by the detailed history of a considerable number of cases of severe injury to the extremities. As a collection of cases (more than fifty in number) showing the extent to which severe injuries may be repaired and limbs saved, that by a less conservative treatment would have been doomed to amputation, this work is of much value. Dr. Walter also claims that the most free exposure of wounds to *pure* air is not injurious. On the contrary, he advocates leaving wounds open, and, if need be, adding incisions sufficient to give the most free exit to extravasations of serum, blood, or pus. The author has made a valuable addition to the surgical literature of the profession.

